# Gevrey-smoothness of lower dimensional hyperbolic invariant tori for nearly integrable symplectic mappings

**DOI:** 10.1186/s13660-017-1307-1

**Published:** 2017-02-07

**Authors:** Shunjun Jiang

**Affiliations:** College of Sciences, Nanjing Tech University, Nanjing, 210009 China

**Keywords:** symplectic mappings, KAM iteration, Gevrey-smoothness, normal form

## Abstract

This paper provides a normal form for a class of lower dimensional hyperbolic invariant tori of nearly integrable symplectic mappings with generating functions. We prove the persistence and the Gevrey-smoothness of the invariant tori under some conditions.

## Introduction and main results

Area-preserving mappings have some dynamical properties similar to Hamiltonian systems, and hence become an important test ground of all kinds of theories for studying Hamiltonian systems, such as Poincaré [1, 2] on three body problem, Moser [3] on the differentiable form of KAM theory, Aubry and Mather [4–8] on Aubry-Mather theorem, Conley and Zehnder [9, 10] on symplectic topology. So area-preserving mappings have attracted many scholars’ interest. We refer to [11–17]. Among all the mappings, symplectic mappings are special for their symplectic structures; we refer to [18–21] for more results on symplectic structures.

On the other hand, many mathematicians turn to the study of the connection between the KAM tori and the parameter. The first work is due to Pöschel [22] who proved that the persisting invariant tori are $C^{\infty}$-smooth in the frequency parameter. Popov [23] obtained the Gevrey-smoothness, a notion intermediate between $C^{\infty}$-smoothness and analyticity, of invariant tori in the frequencies under the Kolmogorov non-degeneracy condition. Xu and You [24] obtained a similar result under the Rüssmann non-degeneracy condition by an improved KAM method. For more results, we refer to [25, 26].

Motivated by [19, 24], we consider the persistence and the Gevrey-smoothness of lower dimensional hyperbolic invariant tori for symplectic mappings determined by generating functions under Rüssmann’s non-degeneracy condition. We consider the following parameterized symplectic mapping:

$$\Phi(\cdot;\xi): (x,u,y, v)\in\mathbb{T}^{n}\times\mathcal{W} \times \mathcal{O} \times\mathcal{W} \to(\hat{x},\hat{u},\hat {y}, \hat{v})\in \mathbb{T}^{n}\times \mathbb{R}^{m}\times\mathbb{R}^{n} \times\mathbb{R}^{m}, $$

where $\xi\in\Pi\subset\mathcal{O}$ is a parameter and $\mathcal {O} \subset\mathbb{R}^{n}$ is a bounded closed connected domain. Suppose $\Phi(\cdot;\xi)$ is implicitly defined by

1.1 $$ \textstyle\begin{cases} \hat{x} =\partial_{\hat{y}}H(x, u, \hat{y},\hat{v};\xi),\\ y =\partial_{x}H(x, u, \hat{y},\hat{v};\xi),\\ \hat{u} =\partial_{\hat{v}}H(x, u, \hat{y},\hat{v};\xi),\\ v =\partial_{u}H(x, u, \hat{y},\hat{v};\xi), \end{cases} $$

where

1.2 $$\begin{aligned}& H(x,u,\hat{y},\hat{v};\xi)= N+P, \end{aligned}$$

1.3 $$\begin{aligned}& N(x, u, \hat{y},\hat{v};\xi)= \bigl\langle x+\omega(\xi), \hat{y} \bigr\rangle + \langle Au,\hat{v}\rangle+\frac{1}{2}\langle B u, u \rangle+\frac{1}{2} \langle C\hat{v}, \hat{v}\rangle. \end{aligned}$$

Suppose *A* is a constant matrix and $B,C$ are symmetric. If $P=0$, Φ can be expressed explicitly as

1.4 $$ \begin{aligned} &\hat{x}=x+\omega(y),\qquad \hat{y}= y, \\ &\hat{u}= \bigl(A-C \bigl(A^{T} \bigr)^{-1}B \bigr)u+C{ \bigl(A^{T} \bigr)^{-1}} v, \qquad \hat{v}=-{ \bigl(A^{T} \bigr)^{-1}} B u+{ \bigl(A^{T} \bigr)^{-1}} v . \end{aligned} $$

We define

$$ \Omega= \left ( \textstyle\begin{array}{@{}c@{\quad}c@{}} A-C(A^{T})^{-1}B&\quad C(A^{T})^{-1} \\ -(A^{T})^{-1}B& \quad(A^{T})^{-1} \end{array}\displaystyle \right )_{2m\times2m}. $$

Denote the eigenvalues of Ω by $(\lambda_{1},\lambda _{2},\ldots,\lambda_{2m})$. We call the lower dimensional invariant torus elliptic if $\vert \lambda_{i} \vert =1$, $\lambda_{i}\neq1$, $\forall i=1,2,\ldots,2m $ and hyperbolic if $\vert \lambda_{i} \vert \neq1, \forall i=1,2,\ldots,2m$.

We note that, although some results on symplectic mappings can be anticipated by Hamiltonian systems, there are still many differences for lower dimensional invariant tori. The first one is concerned with the relations of variables. In symplectic mappings, some variables determined by generating functions take on an implicit form and hence lead to more difficulties than in a Hamiltonian system. The second one is the non-degeneracy condition, which will result in a more complicated proof for estimate of measure.

Before presenting the main result, we give some assumptions and definitions.

### Assumption 1

Rüssmann’s non-degeneracy condition

There exists an integer $\bar{n}>1$ such that

1.5 $$ \operatorname{rank} \bigl\{ \partial^{\beta}_{\xi}\omega(\xi): 1\leqslant \vert \beta \vert \le\bar{n} \bigr\} =n,\quad \forall \xi \in\Pi, $$

where

$$\partial^{\beta}_{\xi}\omega(\xi)= \bigl( \partial^{\beta}_{\xi}\omega _{1}(\xi), \partial^{\beta}_{\xi}\omega_{2}(\xi),\ldots,\partial ^{\beta}_{\xi}\omega_{n}(\xi) \bigr)^{T} $$

with

$$\partial^{\beta}_{\xi}\omega_{i}(\xi)= \frac{\partial^{\vert \beta \vert }_{\xi}\omega_{i}(\xi)}{\partial\xi_{1}^{\beta_{1}}\partial\xi _{1}^{\beta_{2}}\cdots\partial\xi_{n}^{\beta_{n}} }, $$

and

$$\beta=(\beta_{1},\beta_{2},\ldots,\beta_{n}). $$

### Assumption 2

Hyperbolic condition

Let $A=\operatorname{diag}(a_{1},a_{2},\ldots,a_{m}), B=\operatorname{diag}(b_{1},b_{2},\ldots,b_{m})$, $C=\operatorname{diag}(c_{1},c_{2},\ldots,c_{m})$. Define $\Delta_{i}=\frac {a_{i}^{2}-b_{i}c_{i}+1}{a_{i}},i=1,2,\ldots,m$. Suppose $\Delta_{i}^{2}>4,i=1,2,\ldots,m$.

### Remark 1.1

By direct calculation, we have the eigenvalues of Ω,

$$\lambda_{i}=\frac{\Delta_{i}\pm\sqrt{( \Delta _{i})^{2}-4}}{2},\quad i=1,2,\ldots,m. $$

If $\Delta_{i}^{2}>4$, we have $\vert \lambda_{i} \vert \neq1, i=1,2,\ldots ,m$, so the lower dimensional invariant torus is hyperbolic. If otherwise $\Delta_{i}^{2}<4$, we have $\vert \lambda_{i} \vert =1$, and hence the lower dimensional invariant torus is elliptic.

### Definition 1.1

Let $\mathcal{O} \subset\mathbb{R}^{n}$ be a bounded closed connected domain. A function $F:\mathcal {O}\rightarrow\mathbb{R}$ is said to belong to Gevrey-class $G^{\mu}(\mathcal{O})$ of index *μ* ($\mu\geq1$) if *F* is $C^{\infty}(\mathcal{O})$-smooth and there exists a constant *J* such that for all $p\in\mathcal{O}$,

$$\bigl\vert \partial^{\beta}_{p}F(p) \bigr\vert \leq cJ^{\vert \beta \vert +1}\beta!^{\mu}, $$

where $\vert \beta \vert =\beta_{1}+\beta_{2}+\cdots+\beta_{n}$ and $\beta !^{\mu}=\beta_{1}!\beta_{2}!\cdots\beta_{n}! $ for $\beta=(\beta_{1},\beta_{2},\ldots,\beta_{n})\in\mathbb{Z}^{n}_{+}$.

### Remark 1.2

By definition, it is easy to see that the Gevrey-smooth function class $G^{1}$ coincides with the analytic function class. Moreover, we have

$$G^{1}\subset G^{\mu_{1}} \subset G^{\mu_{2}} \subset C^{\infty} $$

for $1<\mu_{1}<\mu_{2}<\infty$.

Set

$$\begin{aligned}& \mathcal{T}_{s}= \bigl\{ x \in\mathbb{C}^{n}/2\pi \mathbb{Z}^{n}: \vert \operatorname{Im}x \vert _{\infty}\le s \bigr\} , \\& \mathcal{B}_{r}= \bigl\{ y\in\mathbb {C}^{n}:\vert y \vert _{1}\le r^{2} \bigr\} , \end{aligned}$$

and

$$\mathcal{W}_{r}= \bigl\{ w\in\mathbb{C}^{m}: \vert w \vert _{2}\le r \bigr\} . $$

Denote by $\mathcal{D}(s,r)=\mathcal{T}_{s}\times\mathcal{W}_{r} \times \mathcal{B}_{r}\times\mathcal{W}_{r}$. Here, $\vert x \vert _{\infty}=\max_{1\le j\le n} \vert x_{j} \vert , \vert y \vert _{1}=\sum_{1\le j\le n} \vert y_{j} \vert $ and $\vert w \vert _{2}= (\sum_{1\le j\le m} \vert w_{j} \vert ^{2} )^{\frac{1}{2}}$.

Denote

$$\Pi= \bigl\{ \xi\in\mathcal{O}\mid \operatorname{dist}(\xi,\partial\mathcal {O} ) \geqslant h \bigr\} $$

and

$$\Pi_{h}= \bigl\{ \xi\in\mathbb{C}^{n}\mid \operatorname{dist}(\xi, \Pi) \leqslant h \bigr\} . $$

### Definition 1.2

$f\in G^{1,\mu}(\mathcal{D}(s,r)\times\Pi)$ means that $f \in C^{\infty}(\mathcal{D}(s,r)\times\Pi) $ and $f(x,y,u,v;\xi) $ is analytic with respect to $(x,y,u,v) $ on $\mathcal{D}(s,r)$ and $G^{\mu}$-smooth in *ξ* on Π.

Below we define some norms. If $P(x,u, \hat{y},\hat{v};\xi)$ is analytic in $(x,u, \hat{y},\hat{v})$ on $\mathcal{D}(s,r)$ and *n̄*-times continuously differentiable in *ξ* on Π, we have

$$P(x,u, \hat{y},\hat{v};\xi)=\sum_{k\in\mathbb{Z}^{n}}P_{k}( u, \hat{y},\hat{v};\xi)e^{\mathrm{i}\langle k,x\rangle}, $$

where

$$P_{k}(u, \hat{y},\hat{v};\xi)=\sum_{l,i,j}P_{klij}( \xi) \hat {y}^{l}u^{i}\hat{v}^{j}. $$

Define

$$\Vert P \Vert _{D(s,r)\times\Pi}=\sum_{k\in\mathbb{Z}^{n}} \vert P_{k} \vert _{r} e^{s\vert k \vert }, $$

where

$$\vert P_{k} \vert _{r}=\sup_{(u,\hat{y}, \hat{v})\in\mathcal{W}_{r}\times\mathcal {B}_{r}\times\mathcal{W}_{r}} \sum_{i,j,l}\Vert P_{klij} \Vert _{s}\hat{y}^{l} u^{i}{\hat{v}}^{j}. $$

This norm is apparently stronger than the super-norm. Moreover, the Cauchy estimate of analytic functions is also valid under this norm.

Let $X_{P}=(-\partial_{\hat{y}}P, -\partial_{\hat{v}}P, \partial_{x}P, \partial_{u}P)$ and denote a weighted norm by

$$\begin{aligned} \Vert X_{P} \Vert _{r;\mathcal{D}(s, r)\times\Pi} ={}&\Vert \partial_{\hat{y}}P \Vert _{D(s,r)\times\Pi} +\frac{1}{r} \Vert \partial_{\hat{v}}P \Vert _{D(s,r)\times\Pi} \\ &{}+\frac{1}{r^{2}} \Vert \partial_{x}P \Vert _{D(s,r)\times\Pi}+\frac{1}{r} \Vert \partial_{u}P\Vert _{D(s,r)\times\Pi}, \end{aligned}$$

where

$$\begin{aligned}& \Vert \partial_{\hat{x}}P \Vert _{D(s,r)\times\Pi} =\sum _{j}\Vert \partial_{\hat{x}_{j}}P \Vert _{D(s,r)\times\Pi}, \\& \Vert \partial_{\hat{y}}P \Vert _{D(s,r)\times\Pi} =\max _{j} \Vert \partial_{\hat{y}_{j}}P\Vert _{D(s,r)\times\Pi} \end{aligned}$$

and

$$\Vert \partial_{u}P \Vert _{D(s,r)\times\Pi}= \biggl(\sum _{j}\bigl(\Vert \partial_{u_{j}}P \Vert _{s,r}\bigr)^{2} \biggr)^{\frac{1}{2}}, $$

$\Vert \partial_{\hat{v}}P \Vert _{D(s,r)\times\Pi} $ is defined similarly.

Now we introduce the main result. Let $\tau\geqslant n \bar{n}-1$. For $\delta\in(0,1)$, let $\mu =\tau+\delta+2$ and $\sigma=(\frac{3}{4})^{\frac{\delta}{\tau +1+\delta}}$.

### Theorem 1.1

*Consider the symplectic mapping* $\Phi(\cdot;\xi)$, *which is implicitly defined by a generating function* $H(\cdot;\xi)$ *in* (1.2). *Let* $\max_{\xi\in\Pi_{h}}\vert \frac{\partial\omega}{\partial\xi} \vert \leqslant T$. *Suppose that Assumptions* 1, 2 *hold*. *Then there exists* $\gamma>0$ *such that for any* $0<\alpha<1$, *if* $\Vert X_{P} \Vert _{r;\mathcal{D}(s,r)\times\Pi _{d}}=\epsilon\leqslant\gamma^{3} \alpha^{2\bar{\nu}}\rho^{2\nu }$ *with* $\bar{\nu}=\bar{n}+1, \nu=\tau(\bar{n}+1)+n+\bar{n}$, *the following results hold true*:

(i) *There exist a non*-*empty Cantor*-*like subset* $\Pi_{*} \subset \Pi$ *and*, *for* $\xi\in\Pi_{*}$, *a symplectic mapping* $\Psi_{*}(\cdot; \xi)$, *where* $\Psi_{*}\in G^{1,\mu}$ *with*
  1.6 $$ \bigl\Vert \partial^{\beta}_{\xi}( \Psi_{*}-\operatorname{id}) \bigr\Vert _{r;D(\frac {s}{2},\frac{r}{2})\times\Pi_{*}}\leq c \rho^{\nu}J^{\vert \beta \vert } \beta!^{\mu}\gamma^{\frac{9}{4(n+1)}} $$
  *for* $\forall\beta\in Z^{+}_{n}$ *and* $J= \frac{2T+1}{\alpha}[ \frac {4(\mu-1)(n+1)}{3}]^{\mu-1}$. *Moreover*, $\Phi_{*}=\Psi_{*}^{-1}\circ \Phi\circ\Psi_{*}$ *is generated by* $H_{*}=N_{*} +P_{*}$ *as in* (1.1) *satisfying*
  $$\begin{aligned}& N_{*}(x, u, \hat{y}, \hat{v};\xi)=\langle x+\omega_{*},\hat{y}\rangle+ \langle A_{*}u, \hat{v}\rangle+\frac{1}{2} \langle B_{*}u, u\rangle+\frac{1}{2} \langle C_{*}\hat{v}, \hat{v}\rangle, \\& P_{*}( x, u,\hat{y}, \hat{v}; \xi)=\sum_{\vert i \vert +\vert j \vert +2\vert l \vert \ge 3}P_{lij}(x; \xi)\hat{y}^{l}u^{i}\hat{v}^{j}. \end{aligned}$$
(ii) *Hence*, *for* $\xi\in\Pi_{*}$, *the symplectic mapping* $\Phi(\cdot; \xi)$ *admits a lower dimensional invariant torus*
  $$T_{\xi}=\Psi_{*} \bigl(T^{n},0,0,0;\xi \bigr), $$
  *whose frequencies* $\omega_{*}$ *satisfy*
  1.7 $$ \bigl\vert \partial^{\beta}_{\xi} \bigl( \omega_{*}(\xi)-\omega(\xi) \bigr) \bigr\vert \leq c\rho ^{2\nu}J^{\vert \beta \vert }\beta!^{\mu}\gamma^{\frac{9}{4(n+1)}} $$
  *and*
  1.8 $$ \bigl\vert \bigl\langle \omega_{*}(\xi),k \bigr\rangle +2\pi l \bigr\vert \geq\frac{\alpha }{(1+\vert k \vert )^{\tau}} $$
  *for all* $\xi\in\Pi_{*}$, $0 \neq k\in Z^{n}$. *Moreover*, *we have*
  $$\operatorname{meas} (\Pi\setminus \Pi_{*})\leqslant c\alpha^{\frac{1}{m}}. $$

## The proof of main results

We will use the idea for Hamiltonian systems in [24] to prove our results. In Section 2.1, one KAM step iteration is presented. The key lies in solving a homological equation. Then we will show the KAM step can iterate infinitely in Section 2.2. Convergence of the iteration and the estimate of measure will be presented in Sections 2.3 and 2.4.

### KAM-step

#### Iteration Lemma

*Consider a symplectic mapping* $\Phi (\cdot;\xi)$ *defined in Theorem* 1.1. *Let* $0< E<1, 0<\rho=(1-\sigma)s/10<\frac{s}{5}$ *and* $0<\eta<\frac{1}{8}$. *Let* $K>0$ *satisfy* $\eta^{2}e^{-K\rho}=E$. *Let*

$$\max_{\xi\in\Pi_{h}} \biggl\vert \frac{\partial\omega}{\partial\xi} \biggr\vert \leqslant T,\qquad h=\frac{\alpha}{2(1+K)^{\tau+1}T}. $$

*Moreover*, $\omega(\xi)$ *satisfies that*: *for* $k\in\mathbb{Z}^{n}\setminus\{0\}$, $l \in\mathbb{Z}$,

2.1 $$ \bigl\vert \langle k, \omega\rangle+2\pi l \bigr\vert \ge \frac{2\alpha}{(1+\vert k \vert )^{\tau}}. $$

*Suppose Assumptions* 1, 2 *hold*. *Suppose that* *P* *satisfies*

$$\Vert X_{P} \Vert _{r;D(s,r)\times\Pi_{d}}\leqslant\epsilon= \eta^{2}\alpha ^{2\bar{\nu}} \rho^{2\nu}E , $$

*with* $0<\alpha<1, \bar{\nu}=\bar{n}+1, \nu=\tau(\bar{n}+1)+n+\bar{n}$. *Then we have the following results*:

1. $\forall\xi\in\Pi_{h}$, *there exists a symplectic diffeomorphism* $\Psi(\cdot;\xi)$ *with*
  $$\begin{aligned}& \Vert \Psi-\operatorname{id} \Vert _{r;D(s-3\rho, \frac{r}{4})\times\Pi_{h}}\leq\frac{c \epsilon}{\alpha^{\bar{\nu}} \rho^{\nu}}, \\& \Vert D \Psi-\operatorname{id} \Vert _{r;D(s-3\rho, \frac{r}{4})\times\Pi_{h}}\leq \frac {c \epsilon}{\alpha^{\bar{\nu}} \rho^{\nu+1}}, \end{aligned}$$
  *such that the conjugate mapping* $\Phi_{+}(\cdot;\xi)=\Psi ^{-1}\circ\Phi\circ\Psi$ *is generated by* $H_{+}(\cdot;\xi)=N_{+}+P_{+}$, *where*
  $$N_{+}= \bigl\langle x+\omega_{+}(\xi),\hat{y} \bigr\rangle + \langle A_{ +}u,\hat{v}\rangle+\frac{1}{2}\langle B_{ +}u,u\rangle+\frac{1}{2}\langle C_{ +}\hat{v}, \hat{v}\rangle $$
  *and* $P_{+}$ *satisfies*
  $$\Vert X_{P} \Vert _{r_{+};D(s_{+},r_{+})\times\Pi_{d}}\leqslant\eta_{+}^{2} \alpha^{2\bar{\nu}}_{+}\rho^{\nu}_{+}E_{+}= \epsilon_{+}, $$
  *with*
  $$\begin{aligned}& s_{+}=s-5\rho,\qquad \rho_{+}=\sigma\rho,\qquad \eta_{+}=E_{+}, \\& r_{+}= \eta r,\qquad E_{+}=E^{\frac{4}{3}},\qquad \frac{\alpha}{2} \leqslant \alpha_{+} \leqslant\alpha. \end{aligned}$$
  *Furthermore*, *we have*
  2.2 $$ \bigl\vert \omega_{+}(\xi)-\omega(\xi) \bigr\vert \leq\epsilon,\quad\forall\xi\in\Pi_{h}. $$
2. *Let* $\alpha_{+}=\alpha-(K+1)^{\tau+1}\epsilon$,
  2.3 $$ \bar{\Pi}= \biggl\{ \xi\in\Pi: \bigl\vert \bigl\langle k, \omega_{+}(\xi) \bigr\rangle \bigr\vert < \frac{2\alpha_{+}}{(1+\vert k \vert )^{\tau}}, k\in Z^{n}, K< \vert k \vert \leq K_{+} \biggr\} , $$
  *and* $\Pi_{+}=\Pi\setminus\bar{ \Pi}$. *Then*, *for* $\forall\xi\in\Pi_{+},\forall k\in Z^{n}$ *and* $0<\vert k \vert \leq K_{+}$, *we have*
  2.4 $$ \bigl\vert \bigl\langle k, \omega_{+}(\xi) \bigr\rangle \bigr\vert \geqslant \frac{2\alpha_{+}}{(1+\vert k \vert )^{\tau}} , $$
  *where* $K_{+}>0$ *such that* $\frac{ e^{-K_{+}\rho_{+}}}{\eta_{+}^{2}}= E_{+}$.
3. *Let* $T_{+}=T+\frac{6\epsilon}{h}$ *and* $h_{+}=\frac{\alpha _{+}}{2(K_{+}+1)^{\tau+1}T_{+}}$. *If* $h_{+}\leq\frac{5}{6}h$, *we have* $\max_{\xi\in\Pi_{h_{+}}} \vert \frac{\partial\omega_{+}}{\partial\xi} \vert \leq T_{+}$, *where* $\Pi_{h_{+}}$ *is the complex* $h_{+}$-*neighborhood of* $\Pi_{+}$.

*A. The equivalent form of* (*1.2*)*.*

Let

2.5 $$\begin{aligned} P(p, \hat{q})={}&P_{000}(x)+ \bigl\langle P_{100}(x), \hat{y} \bigr\rangle + \bigl\langle P_{010}(x), u \bigr\rangle + \bigl\langle P_{001}(x), \hat{v} \bigr\rangle \\ &{}+ \bigl\langle P_{011}(x)u, \hat{v} \bigr\rangle +\frac{1}{2} \bigl\langle P_{020}(x)u, u \bigr\rangle +\frac{1}{2} \bigl\langle P_{002}(x)\hat{v}, \hat{v} \bigr\rangle \\ &{}+ \sum_{\vert i \vert +\vert j \vert +2\vert l \vert \ge 3}P_{lij}\hat{y}^{l}u^{i}\hat{v}^{j} \end{aligned}$$

with

$$P_{lij}=\frac{\partial^{l+i+j} P}{\partial{\hat{y}}^{l} \partial u^{i}\partial{\hat{v}}^{j} }\bigg\vert _{ u=0, \hat{y}=0, \hat{v}=0}. $$

Let

$$Q(x,u,\hat{v})= \bigl\langle Q_{2}(x)u, \hat{v} \bigr\rangle + \frac{1}{2} \bigl\langle Q_{1}(x)u, u \bigr\rangle + \frac{1}{2} \bigl\langle Q_{3}(x)\hat{v}, \hat{v} \bigr\rangle $$

with $Q_{1}(x)=P_{020}(x), Q_{2}(x)=P_{011}(x), Q_{3}(x)=P_{002}(x)$.

Then we rewrite *H* as

$$H=N+Q+(P-Q), $$

where $N+Q$ is the new main term and $P-Q$ is the new small term.

Now we will study the following function which is equivalent to (1.2):

2.6 $$ H(x,u, \hat{y},\hat{v};\xi)=N(x,u, \hat{y},\hat{v};\xi)+P(x,u, \hat{y}, \hat{v};\xi), $$

where

$$N= \bigl\langle x+\omega(\xi), \hat{y} \bigr\rangle +\langle Au,\hat{v}\rangle + \frac{1}{2}\langle B u, u \rangle+\frac{1}{2}\langle C\hat{v}, \hat{v} \rangle+Q(x,u,\hat{v}) $$

and

$$P= P_{000}(x)+ \bigl\langle P_{100}(x), \hat{y} \bigr\rangle + \bigl\langle P_{010}(x), u \bigr\rangle + \bigl\langle P_{001}(x), \hat{v} \bigr\rangle + \sum_{2\vert l \vert +\vert i \vert +\vert j \vert \geqslant3}P_{lij} \hat{y}^{l}u^{i}\hat{v}^{j}. $$

*B. Generating functions of conjugate mappings.*

For convenience, let $p=(x,u)$ and $q=(y,v)$. *p̂* and *q̂* have a similar meaning. The symplectic structure becomes $dp\wedge dq$ on $\mathbb{R}^{n+m}\times \mathbb{R}^{n+m}$. Consider a symplectic mapping $\Phi:(p, q)\rightarrow(\hat{p},\hat{q})$ generated by

2.7 $$ \hat{p}=\partial_{\hat{q}} H(p,\hat{q}), \qquad q=\partial_{p} H(p,\hat{q}), $$

where $H(p,\hat{q})= N(p,\hat{q})+P(p, \hat{q})$, where *N* is the main term and *P* is a small perturbation.

We need a symplectic transformation $\Psi:(p_{+}, q_{+})\rightarrow(p, q)$ generated by

2.8 $$ q=q_{+}+F_{1}(p,q_{+}),\qquad p_{+}=p+F_{2}(p,q_{+}), $$

with

$$\begin{aligned}& F_{1}(p,q_{+})=\frac{\partial F(p,q_{+})}{\partial p}, \\& F_{2}(p,q_{+})= \frac {\partial F(p,q_{+})}{\partial q_{+}}. \end{aligned}$$

The generating function is $\langle p, q_{+}\rangle+F(p, q_{+})$ with *F* being a small function.

By (2.7) and (2.8), we have a conjugate mapping $\Phi=\Psi^{-1}\circ\Phi\circ \Psi: (p_{+},q_{+})\rightarrow(\hat{p}_{+},\hat{q}_{+}) $ implicitly by

2.9 $$ \hat{p}_{+}=H_{2}(p,\hat{q})+F_{2}(\hat{p}, {\hat{q}}_{+}), \qquad q_{+}=H_{1}(p,\hat{q})-F_{1}(p, q_{+}) $$

with

$$\begin{aligned}& H_{1}(p,\hat{q})=\frac{\partial H(p,\hat{q})}{\partial p}, \\& H_{2}(p,\hat{q})= \frac{\partial H(p,\hat{q})}{\partial\hat{q}}. \end{aligned}$$

So we have the following lemma.

#### Lemma 2.1

[19]

*The conjugate symplectic mapping* $\Phi_{+}$ *can be implicitly determined by a generating function* $H_{+}(p_{+}, {\hat{q}}_{+})$ *through*

2.10 $$ \hat{p}_{+}=\partial_{\hat{q}_{+}}H_{+}(p_{+},\hat{q}_{+}),\qquad q_{+}=\partial_{p_{+}} H_{+}(p_{+},\hat{q}_{+}), $$

*where*

2.11 $$ \begin{aligned} H_{+}(p_{+}, {\hat{q}}_{+})={}&H(p, \hat{q})+H_{1}(p,\hat{q})F_{2}(p, q_{+})-H_{2}(p,\hat{q})F_{1}(\hat{p}, {\hat{q}}_{+}) \\ &{}+F(\hat{p}, {\hat{q}}_{+})-F(p,q_{+})-F_{1}(p,q_{+})F_{2}(p,q_{+}), \end{aligned} $$

*where* $p,\hat{p}, \hat{q}, q_{+}$ *depend on* $(p_{+},\hat{q}_{+})$ *as explained above*.

*Set* $ z=(p_{+}, \hat{q}_{+})$. *We have*

2.12 $$ H_{+}(z)=H(z)+F \bigl(N_{2}(z),\hat{q}_{+} \bigr)-F \bigl(p_{+}, N_{1}(z) \bigr)+\Upsilon(z) $$

*with*

$$N_{1}(z)=\frac{\partial N(p_{+},\hat{q}_{+})}{\partial p_{+}}, \qquad N_{2}(z)=\frac{\partial N(p_{+},\hat{q}_{+})}{\partial\hat{q}_{+}} $$

*and* $\Upsilon(z) $ *satisfying*

2.13 $$ |\!|\!| X_{\Upsilon} |\!|\!| _{r;\mathcal{D}(s-5\rho, r/16)}\le \frac{c\epsilon^{2}}{\alpha^{2\bar{\nu}}\rho^{2\nu}}, $$

*where* $\bar{\nu}=\bar{n}+1$ *and* $\nu=\tau(\bar{n}+1)+\bar{n}+n$.

*C. Truncation.*

Let

2.14 $$ P=R+ (P-R), $$

where

2.15 $$\begin{aligned} R(p, \hat{q})&=\sum_{\vert k \vert \leqslant K} \bigl(P^{k}_{000}(x)+ \bigl\langle P^{k}_{100}(x), \hat{y} \bigr\rangle + \bigl\langle P^{k}_{010}(x), u \bigr\rangle + \bigl\langle P^{k}_{001}(x), \hat{v} \bigr\rangle \bigr) \end{aligned}$$

2.16 $$\begin{aligned} & =R_{000}(x)+ \bigl\langle R_{100}(x), \hat{y} \bigr\rangle + \bigl\langle R_{010}(x), u \bigr\rangle + \bigl\langle R_{001}(x), \hat{v} \bigr\rangle , \end{aligned}$$

and

$$P-R=\sum_{\vert l \vert +\vert i \vert +\vert j \vert =1}P_{lij} \hat{y}^{l}u^{i}\hat{v}^{j}+\sum _{2\vert l \vert +\vert i \vert +\vert j \vert \geqslant3}P_{lij}\hat{y}^{l}u^{i} \hat{v}^{j}. $$

Let $F(p, \hat{q})$ possess the same form as (2.15).

*D. Extending the small divisor estimate.*

$\forall\xi\in\Pi_{h}$, there exists $\xi_{0} \in\Pi$ such that $\vert \xi-\xi_{0} \vert < h$. So we have, for $0<\vert k \vert \leqslant K$,

$$\begin{aligned} \bigl\vert \bigl\langle k,\omega(\xi) \bigr\rangle +2\pi l \bigr\vert & \geqslant\bigl\vert \bigl\langle k,\omega (\xi_{0}) \bigr\rangle +2 \pi l\bigr\vert - \bigl\vert \bigl\langle k,\omega(\xi)-\omega( \xi_{0}) \bigr\rangle \bigr\vert \geqslant\frac{\alpha}{(1+\vert k \vert )^{\tau}}. \end{aligned}$$

*E. Homological equation.*

By (2.12), it follows that

2.17 $$\begin{aligned}& N(p_{+},\hat{q}_{+})+P( p_{+},\hat{q}_{+})-F \bigl(p_{+}, N_{p}(p_{+}, \hat{q}_{+}) \bigr)+F \bigl(N_{q}(p_{+},\hat{q}_{+}), \hat{q}_{+} \bigr)+\Upsilon(z) \\& \quad = \bar{N}(p_{+},\hat{q}_{+})+\bar{P}(p_{+},\hat{q}_{+}). \end{aligned}$$

For simplicity, below we drop the subscripts ‘+’ in $p_{+}$ and $\hat{q}_{+}$.

Similar to the discussion in [27], we get the homological equations.

2.18 $$ -F \bigl(N_{q}(p,\hat{q}),\hat{q} \bigr)+F \bigl(p, N_{p}(p,\hat{q}) \bigr)=R-[R]. $$

To solve (2.18), we need some preparations.

Let $x+\omega=\tilde{x} $. Since

$$\hat{p}=N_{\hat{q}}(p, \hat{p})= \bigl(\tilde{x} , (A+Q_{2})u+(C+Q_{3})v \bigr) $$

and

$$q=N_{p}(p, \hat{q})= \bigl(\hat{y}+Q_{x},(A+Q_{2}) \hat{v}+(B+Q_{1})u \bigr), $$

we have

2.19 $$\begin{aligned} F \bigl(p, N_{p}(p, \hat{q}) \bigr) = {}& F_{000}(x)+ \bigl\langle F_{100}(x), \hat{y}+Q_{x} \bigr\rangle + \bigl\langle F_{010}(x), u \bigr\rangle \\ & {}+ \bigl\langle F_{001}(x), (A+Q_{2})^{T} \hat{v}+(B+Q_{1})u \bigr\rangle \end{aligned}$$

and

2.20 $$\begin{aligned} F \bigl(N_{\hat{q}}(p, \hat{q}), \hat{q} \bigr) = {}& F_{000}(\tilde{x})+ \bigl\langle F_{100}( \tilde{x}), \hat{y} \bigr\rangle + \bigl\langle F_{001}(\tilde{x}), \hat{v} \bigr\rangle \\ & {}+ \bigl\langle F_{010}(\tilde{x}), (A+Q_{2})^{T}u+(C+Q_{3}) \hat{v} \bigr\rangle . \end{aligned}$$

So we get

$$F \bigl(N_{\hat{q}}(p,\hat{q}),\hat{q} \bigr)-F \bigl(p, N_{p}(p, \hat{q}) \bigr)=L_{0}+L_{10}+L_{01}- \bigl\langle F_{100}(x), Q_{x} \bigr\rangle , $$

where

$$\begin{aligned}& L_{0}= \bigl(F_{000}(\tilde{x} )- F_{000}(x ) \bigr)+ \bigl\langle F_{100}(\tilde{x})- F_{100}(x), \hat{y} \bigr\rangle , \\& L_{10}= \bigl\langle A^{T}F_{010}(\tilde{x})-F_{010}(x)-BF_{001}(x), u \bigr\rangle + \bigl\langle Q_{2}^{T}F_{010}(\tilde{x})-Q^{T}_{1}F_{010}(x), u \bigr\rangle \end{aligned}$$

and

$$L_{01}= \bigl\langle C^{T}F_{010}(\tilde{x})+F_{010}(x)-AF_{001}(x), u \bigr\rangle + \bigl\langle Q_{3}^{T}F_{010}(\tilde{x})-Q_{2}F_{010}(x), u \bigr\rangle . $$

After these preparations, we can solve (2.18) which is equivalent to solving the following:

2.21 $$ \textstyle\begin{cases} F_{000}(\tilde{x} )- F_{000}(x )=R_{000}(x)-[R_{000}], \\ F_{100}(\tilde{x})- F_{100}(x)=R_{100}(x)-[R_{100}], \end{cases} $$

and

2.22 $$ \textstyle\begin{cases} A^{T}F_{010}( \tilde{x})-F_{010}(x)-BF_{001}(x)+ Q_{2}^{T}F_{010}( \tilde{x})-Q^{T}_{1}F_{010}(x)=R_{010}(x), \\ C^{T}F_{010}(\tilde{x})+F_{010}(x)-AF_{001}(x)+ Q_{3}^{T}F_{010}(\tilde{x})-Q_{2}F_{010}(x)=R_{001}(x). \end{cases} $$

Firstly, we solve (2.21) for $F_{000} $ and $F_{100} $. Expand $F_{000}(x)$ and $R_{000}(x)$:

$$\begin{aligned}& F_{000}(x)=\sum_{k\in\mathbb{Z}^{n}} F_{k000}e^{\mathrm{i}\langle k, x\rangle}, \\& R_{000}(x)=\sum _{k\in\mathbb{Z}^{n}} R_{k000}e^{\mathrm{i}\langle k, x\rangle}. \end{aligned}$$

Then we get

2.23 $$ F_{kj00}=\frac{1}{e_{k}-1} R_{kj00} $$

with $e_{k}=e^{\mathrm{i}\langle k, \omega\rangle}, k\ne0$. By Assumption 2, we have the following estimate:

$$\Vert F_{k000} \Vert _{s-\rho}\le\frac{c\Vert R_{k000} \Vert _{s} }{\alpha\rho^{\tau +n}}. $$

So we have

$$\Vert X_{F_{000}} \Vert _{r;D(s-\rho)\times\Pi}\leq\frac{c\Vert R_{000} \Vert _{s} }{\alpha^{\bar{\nu}}{\rho^{\nu}}} . $$

Similarly we have

$$\Vert X_{F_{100}} \Vert _{r;D(s-\rho)\times\Pi}\leq\frac{c\Vert R_{100} \Vert _{s} }{\alpha^{\bar{\nu}} \rho^{\nu}}. $$

Next we will get $F_{010}$ and $F_{001}$ from (2.22). Let $F_{010}=(F^{1}_{010}, \ldots, F^{m}_{010})$ and $F_{001}=(F^{1}_{001}, \ldots, F^{m}_{001})$. Expand $F^{l}_{0i'j'}(x)$ and $R^{l}_{0i'j'}(x)$:

$$\begin{aligned}& F^{l}_{0i'j'}(x)=\sum_{k\in\mathbb{Z}^{n}} F^{l}_{k0i'j'}e^{\mathrm {i}\langle k, x\rangle}, \\& R^{l}_{0i'j'}(x)= \sum_{k\in\mathbb{Z}^{n}} R^{l}_{k0i'j'}e^{\mathrm{i}\langle k, x\rangle} \end{aligned}$$

with $l=1,2,\ldots,m$ and $(i',j')=(0,1),(1,0)$.

Let

$$\begin{aligned}& X= \left ( \textstyle\begin{array}{@{}c@{}} F^{l}_{010} \\ F^{l}_{001} \end{array}\displaystyle \right ), \qquad Y=\left ( \textstyle\begin{array}{@{}c@{}} R^{l}_{010} \\ R^{l}_{001} \end{array}\displaystyle \right ), \\& M_{k}=\left ( \textstyle\begin{array}{@{}c@{\quad}c@{}} ae_{k}-1 & -b \\ be_{k}& e_{k}-a \end{array}\displaystyle \right ). \end{aligned}$$

To get the estimate of $F^{l}_{0i'j'}(x)$, we rewrite (2.22) as the following form:

2.24 $$ \sum_{k\in Z^{n}} M_{k}X_{k}e^{\mathrm{i}\langle k, x\rangle}= \sum_{k\in Z^{n}}Y_{k}e^{\mathrm{i}\langle k, x\rangle}+\sum _{k\in Z^{n}} N_{k}X_{k}e^{\mathrm{i}\langle k, x\rangle}, $$

where $N_{k}$ is composed of the components of $Q_{j}, j=1,2,3,4$. We can set $\vert N_{k} \vert \leqslant\epsilon_{0}$.

By a direct calculation, we have

$$\vert M_{k} \vert = \vert e_{k}- \lambda_{i}\vert \bigl\vert e_{k}-\lambda'_{i} \bigr\vert , $$

where

$$\lambda_{i}=\frac{ \Delta_{i}+\sqrt{( \Delta _{i})^{2}-4}}{2},\qquad \lambda_{i}= \frac{ \Delta_{i}-\sqrt{( \Delta _{i})^{2}-4}}{2}, \quad i=1,2,\ldots,m, $$

are the eigenvalues of Ω. By Assumption 2, we have $\vert \lambda_{i} \vert \neq1, \vert \lambda'_{i} \vert \neq 1$. Since $\vert e_{k} \vert =1$, it follows that $\vert M_{k} \vert >c_{0}>0$. We rewrite (2.24) as

$$\Lambda X=Y+\Lambda_{1} X. $$

Since $\vert M_{k} \vert >0$, we have the operator Λ is invertible and hence $X=\Lambda^{-1}(Y+\Lambda_{1}X)$. Set $\Xi X=X-\Lambda^{-1}\Lambda_{1}X$, then we have $\Xi X=L^{-1}Y$. So

$$\begin{aligned} \Vert \Xi X_{1}- \Xi X_{2} \Vert &=\bigl\Vert \Lambda^{-1}\Lambda_{1}X_{1}-\Lambda^{-1} \Lambda_{1}X_{1} \bigr\Vert \\ & \leqslant \bigl\Vert \Lambda^{-1} \bigr\Vert \cdot\bigl\Vert \Lambda _{1}\Vert \cdot \Vert X_{1}-X_{2} \bigr\Vert . \end{aligned}$$

Set $\epsilon_{0}=\frac{c_{0}}{2}$, then we have

$$\Vert \Xi X_{1}- \Xi X_{2} \Vert \leqslant \frac{1}{2}\Vert X_{1}-X_{2} \Vert . $$

By the implicit function theorem, we have $\Vert X \Vert \leqslant c\Vert Y \Vert $, with *c* depending on $A,B,C$. So

$$\Vert F_{0i'j'} \Vert _{D(s-\rho,r)\times\Pi}\le\frac{c r\Vert R_{0i'j'} \Vert _{D(s,r)\times\Pi}}{\alpha^{\bar{\nu}} \rho^{\nu}} $$

with $(i',j')=(0,1),(1,0)$.

From the above discussion, we have

2.25 $$ \Vert X_{F} \Vert _{r;D(s-\rho,r)\times\Pi}\le \frac{c}{\alpha^{\bar{\nu}}\rho^{\nu}} \Vert X_{R} \Vert _{r;D(s,r)\times\Pi}\le \frac{c\epsilon}{\alpha^{\bar{\nu}} \rho^{\nu}}, $$

where $\bar{\nu}=\bar{n}+1$ and $\nu=\tau(\bar{n}+1)+\bar{n}+n$.

By (2.8) and (2.25), we obtain

$$\begin{aligned}& \Vert \Psi-\operatorname{id} \Vert _{r;D(s-3\rho, \frac{r}{4})\times\Pi}\leq\frac{c \epsilon}{\alpha^{\bar{\nu}} \rho^{\nu}}, \\& \Vert D \Psi-\operatorname{id} \Vert _{r;D(s-3\rho, \frac{r}{4})\times\Pi}\leq \frac{c \epsilon}{\alpha^{\bar{\nu}}\rho^{\nu+1}}. \end{aligned}$$

From the above discussion, we get the conjugate mapping $\Phi _{+}(\cdot;\xi)= \Psi^{-1}\circ\Phi\circ\Psi$ generated by $H_{+}=\bar{N}+\bar{P}$, where

$$\bar{N}=[R_{000}]+ \bigl\langle x+\omega(\xi)+R_{100}, \hat{y} \bigr\rangle +\langle Au,\hat{v}\rangle+\frac{1}{2}\langle B u, u \rangle+ \frac{1}{2}\langle C\hat{v}, \hat{v}\rangle+Q(x,u,\hat{v}), $$

and

$$\bar{P}=\Upsilon+(P-R)- \bigl\langle F_{100}(x), Q_{x} \bigr\rangle . $$

Recalling (2.6), we find there are second order terms of $u,\hat{v}$ in $P_{+}$, so we will put these terms into the main term. Let $Q_{+}=-\langle F_{100}(x), Q_{x}\rangle+\Upsilon_{1}$, where $\Upsilon_{1}$ contains the second order term on $u,\hat{v}$ in ϒ.

Then we get $H_{+}=N_{+}+P_{+}$, where

$$\begin{aligned} N_{+}={}& [R_{000}]+ \bigl\langle x+\omega( \xi)+R_{100}, \hat{y} \bigr\rangle +\langle Au,\hat{v}\rangle+\frac{1}{2}\langle B u, u \rangle+\frac{1}{2}\langle C\hat{v}, \hat{v}\rangle+Q(x,u,\hat{v})+ Q_{+} \end{aligned}$$

and

$$P_{+}=(P-R)+\Upsilon-\Upsilon_{1}. $$

We note that $N_{+}$ has the same form as *N*.

Since

$$P-R=\sum_{\vert l \vert +\vert i \vert +\vert j \vert =1}P_{lij} \hat{y}^{l}u^{i}\hat{v}^{j}+\sum _{2\vert l \vert +\vert i \vert +\vert j \vert \geqslant3}P_{lij}\hat{y}^{l}u^{i} \hat{v}^{j}, $$

we have

2.26 $$ \Vert X_{P-R} \Vert _{\eta r; \mathcal{D}(s-5\rho, \eta r)\times\Pi }\leqslant c \cdot \epsilon \biggl(\eta+\frac{e^{-K\rho}}{\eta^{2}} \biggr). $$

By (2.13) and (2.26), we have

$$\Vert X_{P_{+}} \Vert _{\eta r; D(s-5\rho, \eta r)\times\Pi}\le c\cdot\epsilon \biggl( \eta+\frac{e^{-K\rho}}{\eta^{2}} \biggr) + \frac{c\epsilon^{2}}{\eta^{2}\alpha^{2\bar{\nu}}\rho^{2\nu}} . $$

*F. Choice of parameters.*

We choose $0< E<1$ and set

$$\eta=E, \qquad \epsilon=\eta^{2}\alpha^{2\overline{\nu}}\rho^{2\nu }E,\qquad\frac{e^{-K\rho}}{\eta^{2}}=E,\qquad h=\frac{\alpha}{2(K+1)^{\tau+1}T}. $$

Fix $\sigma\in(0,1)$. We define

$$\begin{aligned}& \rho_{+}=\sigma\rho, \qquad s_{+}=s-5\rho,\qquad r_{+}=\eta r, \\& \alpha_{+}=\alpha-(K+1)^{\tau+1}\epsilon,\qquad \epsilon_{+}=c\eta\epsilon,\qquad E_{+}=cE^{\frac{4}{3}}. \end{aligned}$$

By the estimate of $P_{+}$, supposing $\alpha<2\alpha_{+}$, we have

$$\begin{aligned} \Vert X_{P_{+}} \Vert _{\eta r; D(s-5\rho, \eta r)\times\Pi_{+}} &\le c\cdot \epsilon \biggl( \eta+\frac{e^{-K\rho}}{\eta^{2}} \biggr) + \frac{c\epsilon^{2}}{\eta^{2}\alpha^{2\bar{\nu}}\rho^{2\nu}} \\ & \leq c\eta\epsilon=c\alpha^{2\overline{\nu}}\rho ^{2\nu}E^{4} \\ & \leq c\alpha^{2\bar{\nu}}_{+}\rho^{2\tau}_{+}E_{+}^{3}. \end{aligned}$$

Setting $\epsilon_{+}=c\alpha_{+}\rho^{\tau}_{+}E_{+}^{3}$, we arrive at

$$\Vert X_{P_{+}} \Vert _{r_{+};\mathcal{D}(s_{+},r_{+})\times\Pi_{+}}\le\epsilon_{+}. $$

By Iteration Lemma, we have

$$\begin{aligned} \bigl\vert \bigl\langle k,\omega_{+}(\xi) \bigr\rangle +2\pi l \bigr\vert &\geqslant\bigl\vert \bigl\langle k,\omega(\xi) +2\pi l \bigr\rangle \bigr\vert - \bigl\vert \bigl\langle k,\omega_{+}(\xi)-\omega (\xi) \bigr\rangle \bigr\vert \\ &\geq\frac{2}{(1+\vert k \vert )^{\tau}} \bigl[\alpha-(1+K)^{\tau+1}\epsilon \bigr] \\ &=\frac{2\alpha_{+}}{(1+\vert k \vert )^{\tau}}, \end{aligned}$$

where $\xi\in\Pi_{+}$ and $0\neq k\leq K$. So we choose $\alpha_{+}= \alpha-(1+K)^{\tau+1}\epsilon$. By the choice of $\alpha_{+}$, the definition of Π̄ in (2.3) and $\Pi_{+}=\Pi\setminus\bar{ \Pi}$, it follows, for $\forall\xi\in\Pi_{+}$,

$$\bigl\vert \bigl\langle k, \omega_{+}(\xi) \bigr\rangle +2\pi l \bigr\vert \geqslant \frac{2\alpha_{+}}{(1+\vert k \vert )^{\tau}},\quad \forall k \in\mathbb{Z}^{n},0< \vert k \vert \leq K_{+}. $$

Now we give the choice of $T_{+}$. Suppose $h_{+}\leq\frac{5}{6}h$. By the Cauchy estimate, for $\xi\in \Pi_{h_{+}}^{+} $, we have

$$\bigl\vert \partial \bigl(\omega_{+}(\xi)-\omega(\xi) \bigr)/ \partial\xi \bigr\vert _{h_{+}}\leq \frac{\vert \omega_{+}(\xi)-\omega(\xi) \vert _{h}}{h-h_{+}}\leq \frac {6\epsilon}{h}. $$

Define $T_{+}=T+\frac{6\epsilon}{h}$ and $h_{+}=\frac{\alpha _{+}}{T_{+}(1+K_{+})^{\tau+1}}$, then we have

$$\max_{\xi\in\Pi_{h_{+}}}\vert \partial\omega_{+}/ \partial\xi \vert \leqslant\max_{\xi\in\Pi_{h_{+}}}\bigl\vert \partial \bigl(\omega_{+}-\omega (\xi) \bigr)/ \partial\xi \bigl\vert +\max _{\xi\in\Pi_{h_{+}}} \vert \partial\omega/ \partial\xi \vert \leq T_{+}. $$

Thus all the parameters for $H_{+}$ are defined, and so Iteration Lemma is proved.

### Iteration

Define inductive sequences

$$\begin{aligned}& \rho_{j+1}=\sigma\rho_{j}, \qquad s_{j+1}=s_{j}-5\rho,\qquad r_{j+1}=\eta_{j} r_{j}, \\& \alpha_{j+1}=\alpha_{j}-(1+K_{j})^{\tau+1}\epsilon _{j},\qquad E_{j+1}=cE_{j}^{\frac{4}{3}},\qquad T_{j+1}=T_{j}+ \frac{6\epsilon_{j}}{d_{j}}, \\& \eta_{j+1}=E_{j+1},\qquad \epsilon_{j+1}= \alpha^{2\bar{\nu}}_{j}\rho _{j+1}^{2\nu}E_{j+1}{ \eta_{j+1}^{2}}, \end{aligned}$$

and

$$\frac{e^{-K_{j+1}\rho_{j+1}}}{\eta _{j+1}^{2}}=E_{j+1},\qquad h_{j+1}=\frac{\alpha_{j+1}}{(1+K)_{j}^{\tau+1}T_{j+1}}. $$

Define

$$\Pi_{j+1}= \biggl\{ \xi\in\Pi_{j}: \bigl\vert \bigl\langle \omega_{j}(\xi)+2\pi l,k \bigr\rangle \bigr\vert \geqslant \frac{2\alpha_{j}}{( \vert k\vert +1)^{\tau}}, K_{j}< \vert k \vert \leq K_{j+1} \biggr\} $$

and

$$\Pi_{h_{j+1}}= \bigl\{ \xi\in C^{n}: \operatorname{dist}( \xi, \Pi _{j+1})\leqslant h_{j+1} \bigr\} . $$

In the following we give some estimates for Gevrey-smoothness.

Let $\gamma_{j}=K_{j}\rho_{j}=-\ln E_{j}^{3}$. We have $\frac {K_{j+1}}{K_{j}}=\frac{1}{2}\frac{\operatorname{ln}c}{\operatorname{ln}E_{j}}+\frac{4}{3\sigma}$, and hence $\frac{4}{3}\leq\frac{K_{j+1}}{K_{j}}\leq\frac{4}{3}\frac{1}{\rho }$ for $E_{0}$ small enough. If $12< K_{j}< K_{j+1}$, we have $\frac{h_{j+1}}{h_{j}}=\frac{\alpha_{j+1}}{\alpha _{j}}\frac{T_{j}}{T_{j+1}}\frac{(1+K_{j})^{\tau}}{(1+K_{j+1})^{\tau }}\leq\frac{5}{6}$, and hence $h_{j+1}\leq\frac{5}{6} h_{j}$, which means $h_{+}\leq \frac{5}{6} h$ holds. Suppose $\max_{ \xi\in\Pi_{h_{j}}}\vert \frac{\partial\omega_{j}}{\partial\xi} \vert \leq T_{j}$. Let $T_{j+1}=T_{j}+\frac{6\epsilon_{j}}{d_{j}}$. Then we have $\vert \frac{\partial\omega_{j+1}}{\partial\xi} \vert =\vert \frac{\partial(\omega_{j+1}- \omega_{j} +\omega_{j})}{\partial\xi} \vert \leq T_{j+1}$. By the choice of *σ*, we can easily get that $\rho_{j+1} \gamma_{j+1}^{\frac{\delta}{\tau+1}}\geq\rho_{j} \gamma_{j}^{\frac{\delta}{\tau+1}}$. Since $\rho_{0} \gamma_{0}^{\frac{\delta}{\tau+1}}\geq1$, we have $\rho_{j} \gamma_{j}^{\frac{\delta}{\tau+1}}\geq1$ for all $j>1$.

By the definitions of $T_{j},h_{j}$ and $\epsilon_{j}$, we have $T_{j+1}=T_{j}+\frac{6\epsilon_{j}}{d_{j}} =T_{0}+6\sum_{i=0}^{j} (\gamma_{i})^{\tau}e^{-\gamma_{i}}T_{i}$. Noting $\gamma_{j}=-\ln E_{j}^{3}$ and $E_{j}\leqslant (cE_{0})^{(\frac{4}{3})^{j}}$, we can choose $E_{0}$ to be sufficiently small such that $\sum_{i=0}^{j} (\gamma_{i})^{\tau}e^{-\gamma_{i}}T_{i}\leq\frac{1}{6}$, then we have $T_{0}\leq T_{j}\leq T_{0}+1$. Similarly, we have $\frac{1}{2}\alpha_{j}\leq\alpha_{j+1}\leq\alpha_{j}$.

By Iteration Lemma, there exists a sequence of symplectic mappings $\{\Psi_{j}(\cdot;\xi)\}$, generated by $\langle p, q_{+} \rangle+ F_{j}(p,q_{+})$, satisfying

$$\begin{aligned}& \Vert \Psi_{j}-\operatorname{id} \Vert _{r_{j};D(s_{j}-3\rho_{j},r_{j})\times\Pi_{h_{j}}}\le \frac{c \epsilon_{j} }{\alpha_{j}^{\bar{\nu}} \rho_{j}^{\nu}}, \\& \Vert D\Psi_{j}-\operatorname{Id} \Vert _{r_{j};D(s_{j}-3\rho_{j},r_{j})\times\Pi_{h_{j}}}\le \frac{c \epsilon_{j} }{\alpha_{j}^{\bar{\nu}} \rho_{j}^{\nu+1}}. \end{aligned}$$

Define $\Psi^{j}=\Psi_{1}\circ\Psi_{2}\circ\cdots\circ\Psi_{j}$. Then we have a sequence of symplectic mappings $\{\Phi_{j+1}(\cdot ;\xi)=(\Psi^{j})^{-1}\circ\Phi_{j}\circ\Psi^{j}\}$, generated by $H_{j+1}(\cdot;\xi)=N_{j+1}+P_{j+1}$, where

$$N_{j+1}= \bigl\langle x+\omega_{j+1}(\xi),\hat{y} \bigr\rangle +\langle A_{ j+1}u,\hat{v}\rangle+\frac{1}{2}\langle B_{ j+1}u,u\rangle+\frac{1}{2}\langle C_{ j +1}\hat{v}, \hat{v}\rangle $$

with

$$\vert \omega_{j+1}-\omega_{j} \vert \le c \epsilon_{j},\quad \forall j \geqslant1, $$

and

$$\Vert X_{P_{j+1}} \Vert _{ r_{j+1}; \mathcal{D}(s_{j+1}, r_{j+1})\times\Pi _{h_{j+1}}}\le\epsilon_{j+1}. $$

### The convergence of the KAM iteration

Now we prove the convergence of the KAM iteration. Similar to [27], we have

$$\bigl\Vert \Psi^{j}-\Psi^{j-1} \bigr\Vert _{r_{j};D(s_{j}-3\rho_{j},r_{j})\times\Pi _{h_{j}}}\leq c \alpha_{j-1}^{\bar{\nu}}\rho_{j-1}^{\nu} E^{3}_{j-1}, $$

and

$$\bigl\Vert D \bigl(\Psi^{j}-\Psi^{j-1} \bigr) \bigr\Vert _{r_{j};D(s_{j}-3\rho_{j},r_{j})\times\Pi _{h_{j}}}\leq\alpha_{j-1}^{\bar{\nu}} \rho_{j-1}^{\nu+1} E^{3}_{j-1}. $$

By the Cauchy estimate, we have

$$\begin{aligned}& \bigl\Vert \partial^{\beta}_{\xi} \bigl(\Psi^{j}- \Psi^{j-1} \bigr) \bigr\Vert _{r_{j};D(s_{j}-3\rho_{j},r_{j})\times\Pi_{h_{j}}}\leq\frac {c\alpha_{j-1}^{\bar{\nu}}\rho_{j-1}^{\nu} E^{3}_{j-1}\beta!}{h_{j}^{\vert \beta! \vert }}, \\& \bigl\Vert \partial^{\beta}_{\xi}D \bigl(\Psi^{j}- \Psi^{j-1} \bigr) \bigr\Vert _{r_{j};D(s_{j}-3\rho_{j},r_{j})\times\Pi_{h_{j}}}\leq\frac {c\alpha_{j-1}^{\bar{\nu}}\rho_{j-1}^{\nu+1} E^{3}_{j-1}\beta !}{h_{j}^{\vert \beta! \vert }} \end{aligned}$$

and

$$\bigl\Vert \partial^{\beta}_{\xi} \bigl(\omega^{j}- \omega^{j-1} \bigr) \bigr\Vert _{\Pi _{j}}\leq \frac{c\epsilon_{j-1}\beta!}{h_{j}^{\vert \beta! \vert }}. $$

Let $U_{j}^{\beta}=\frac{c\alpha_{j-1}^{\bar{\nu}}\rho _{j-1}^{\nu} E^{3}_{j-1}\beta!}{h_{j}^{\vert \beta! \vert }}$ and $G_{j}^{\beta}=\frac{c\epsilon_{j-1}\beta!}{h_{j}^{\vert \beta! \vert }}$. Now we estimate $U_{j}^{\beta} $ and $G_{j}^{\beta}$ for $\beta\in Z^{+}_{n}$.

Since $\rho_{j} \gamma_{j}^{\frac{\delta}{\tau+1}}\geq1$ for all $j>1$, we have $\frac{1}{\rho_{j}}\leq\gamma_{j}^{\frac {\delta}{\tau+1}}$. Then we have $K_{j}=\frac{\gamma_{j}}{\rho_{j}} \leq\gamma _{j}^{1+\frac{\delta}{\tau+1}}$, which means that $K_{j}^{\tau+1}\leq\gamma_{j}^{\tau+1+\delta}$. Noting that $h_{j}= \frac{\alpha_{j}}{2(K+1)_{j}^{\tau+1}T_{j}}, T_{j}< T+1, \frac{1}{2}\alpha\leq\alpha_{j}$ and $E_{j-1}= E_{j}^{\frac{3}{4}}=e^{-\frac{\gamma_{j}}{4}}$, we have

$$\begin{aligned} U_{j}^{\beta}&\leq c \alpha^{\bar{\nu}} \rho_{j}^{\nu} \beta! \biggl( \frac{T+1}{\frac{\alpha}{2}} \biggr)^{\vert \beta \vert } \bigl(\gamma_{j}^{\tau+1+\delta} \bigr)^{\vert \beta \vert }e^{-\frac{3\gamma_{j}}{4}} \\ &\leq c \rho_{j}^{\nu} \biggl( \frac{2(T+1)}{\alpha} \biggr)^{\vert \beta \vert } \beta! \bigl[ \gamma_{j}^{\beta_{1}}e^{-\frac{3\gamma_{j}}{4}\frac {1}{(\tau+\delta)(n+1)}} \cdots\gamma_{j}^{\beta_{n}}e^{-\frac{3\gamma _{j}}{4}\frac{1}{(\tau+\delta)(n+1)}}\bigr]^{\tau+\delta}e^{-\frac {3\gamma_{j}}{4}\frac{1}{n+1}} \\ &\leq c \rho_{j}^{\nu}J^{\vert \beta \vert }\beta!^{\mu}J_{j}^{\frac {9}{4(n+1)}}, \end{aligned}$$

where $J= \frac{2T+1}{\alpha}[ \frac{4(\mu-1)(n+1)}{3}]^{\mu-1},\mu=\tau+\delta$, and *c* only depends on $n,\alpha,\mu$.

In the same way, we have

$$G_{j}^{\beta}\leq c \rho_{j}^{2\nu}J^{\vert \beta \vert } \beta!^{\mu }E_{j}^{\frac{9}{4(n+1)}}. $$

Note that $s_{j}\rightarrow\frac{s}{2}, r_{j}\rightarrow0, h_{j}\rightarrow0$ as $j\rightarrow\infty$. Let $D_{*}=D(\frac{s}{2},0), \Pi_{*}=\bigcap_{j\geq0}\Pi^{j}$ and $\Psi_{*}=\lim_{j\rightarrow\infty}\Psi^{j}$. Then we have

$$\bigl\Vert \partial^{\beta}_{\xi}(\Psi_{*}- \operatorname{id}) \bigr\Vert _{\frac{r}{2};D(*)\times \Pi_{*}}\leq c\rho_{0}^{\nu} J^{\vert \beta \vert }\beta!^{\mu}E_{0}^{\frac {9}{4(n+1)}}, \quad \forall \beta\in Z^{+}_{n}. $$

Since $\Psi_{j}$ is affine in *y*, $\Psi^{j}$ is also affine in *y*, and hence we have the convergence of $\partial^{\beta}_{\xi }\Psi^{j} $ to $\partial^{\beta}_{\xi}\Psi^{*}$ on $D(\frac{s}{2},\frac{r}{2})$ and

$$\bigl\Vert \partial^{\beta}_{\xi}(\Psi_{*}- \operatorname{id}) \bigr\Vert _{\frac{r}{2};D(\frac {s}{2},\frac{r}{2})\times\Pi_{*}}\leq c\rho_{0}^{\nu} J^{\vert \beta \vert }\beta!^{\mu}E_{0}^{\frac{9}{4(n+1)}}, $$

$\forall\beta\in Z^{+}_{n}$. Thus we proved (1.6).

Let $\omega_{*}=\lim_{j\rightarrow\infty}\omega_{j}$. Similarly, it follows that

$$\bigl\vert \partial^{\beta}_{\xi} \bigl(\omega_{*}( \xi)-\omega \bigr) (\xi) \bigr\vert _{\Pi _{*}}\leq c\rho_{0}^{2\nu}J^{\vert \beta \vert } \beta!^{\mu}E_{0}^{\frac{9}{4(n+1)}}, \quad \forall\beta\in Z^{+}_{n}. $$

Moreover we have

$$\bigl\vert \bigl\langle \omega_{*}(\xi),k \bigr\rangle \bigr\vert \geq\frac{\alpha _{*}}{(1+\vert k \vert )^{\tau}} $$

for all $\xi\in\prod_{*}$ and $0 \neq k\in Z^{n}$, where $\alpha_{*}= \lim_{j\rightarrow\infty}\alpha^{j}$, with $\frac{\alpha}{2} \leq\alpha_{*}\leq\alpha$. Thus we proved (1.7) and (1.8).

### Estimate of measure

We note that $\beta\geqslant1$ in Assumption 1 for symplectic mappings , while $\beta\geqslant0$ in Hamiltonian systems [24, 25]. So the non-degeneracy condition in symplectic mappings and that in Hamiltonian systems are different. It means that the estimate of measure is different in two cases. But the proof for symplectic mappings is similar to [24, 25], so we omit the details.
